# Editorial: Sex differences in cancer incidence, mortality, and survival: methodological perspectives

**DOI:** 10.3389/fonc.2024.1441965

**Published:** 2024-07-05

**Authors:** Syed Ahsan Raza, Wilson Luiz da Costa, Aaron P. Thrift

**Affiliations:** ^1^ Department of Surgery, University of Pittsburgh, Pittsburgh, PA, United States; ^2^ Department of Medicine, Baylor College of Medicine, Houston, TX, United States

**Keywords:** cancer, incidence, mortality, survival, sex ratio, burden

Cancer is a leading cause of death in many developed countries and is the major cause of not only mortality but also morbidity in every region of world regardless of the country’s healthcare resources. Based on the principle of epidemiological transition related to aging, changing lifestyles and economic factors, there will be a dramatic world-wide increase in the number of cancers in the next few decades. It has been predicted that the number of incident cases worldwide will be 20.3 million cancer cases by 2030 (Raza et al.).

Sex differences, or the sex ratio (i.e., the male-to-female cancer incidence rate), is a valuable measure for addressing issues of artifacts and imperfect cancer case ascertainment in various cancer registries worldwide. The “Sex-Ratio Methodology” introduced new perspectives in disease epidemiology, especially in cases where the etiology remains unknown or where new hypotheses are needed, while also confirming existing ones. The magnitude of sex ratio is a robust epidemiological marker, and its variability can be used to compare data from different countries and regions across multiple cancer type (**Figure 1** provides an example of methodology on magnitude of sex ratio and it’s variation in specific time period using publicly available dataset from Cancer Incidence in Five Continents). Recently, the sex ratio has been utilized in cancer epidemiology using country-specific or global cancer registries to explore potential causes of cancers across different time periods (Raza et al).

**Figure 1:**
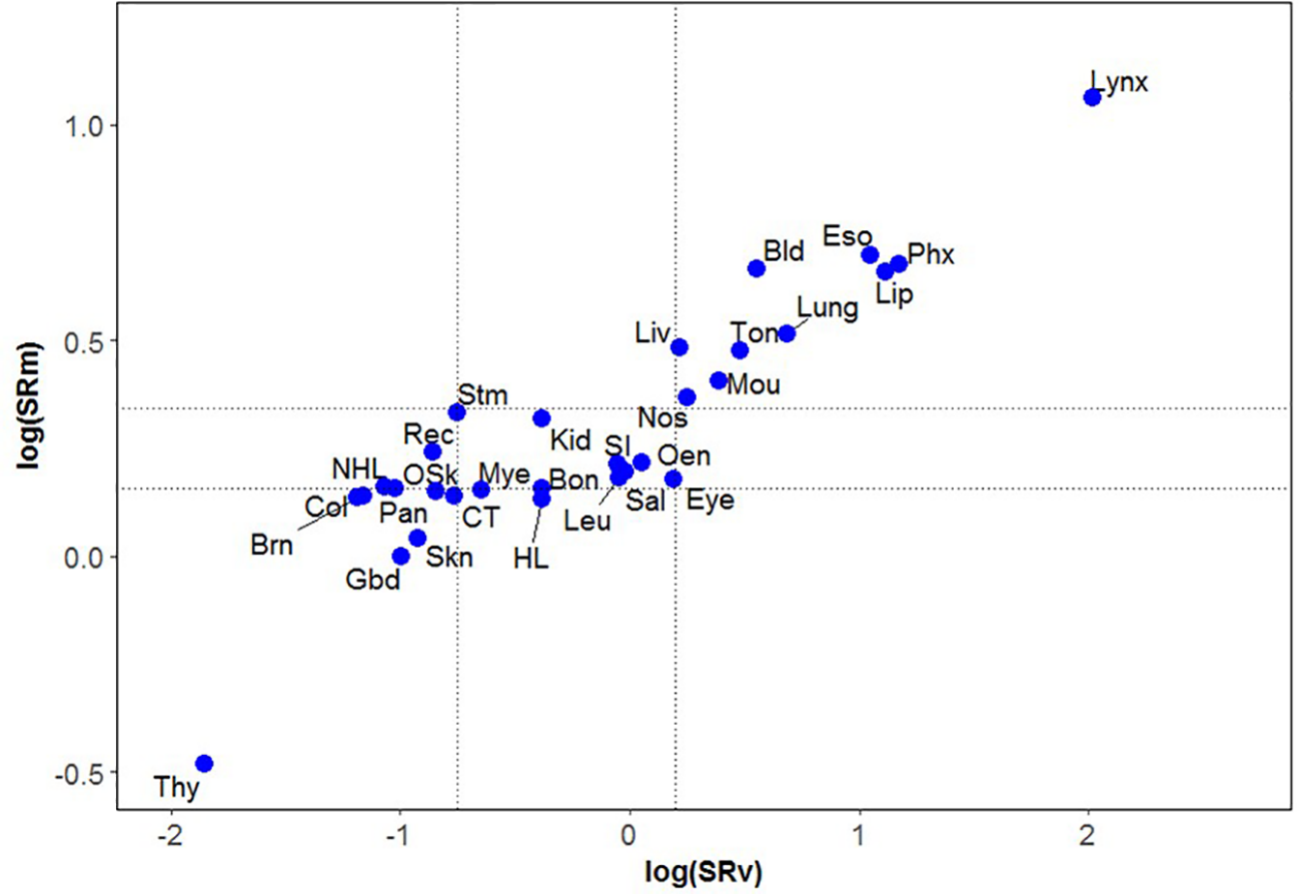
Magnitude of the sex ratios (SRm) of 30 cancer types* plotted against their variances (SRv) on log scale for period 2003-07. Footnote (**Figure 1**). *Bladder (Bld); Bone (Bon); Brain (Brn); Colon (Col); Connective Tissue (CT); Eye (Eye); Gallbladder (Gbd); Hodgkin Lymphoma (HL); Kidney (Kid); Larynx (Lynx); Leukemia (Leu); Lip (Lip); Liver (Liv); Lung (Lung); Melanoma of Skin (Skn); Mouth (Mou); Multiple Myeloma (Mye); Non Hodgkin Lymphoma (NHL); Nose and Sinuses (Nos); Oesophagus (Eso); Other endocrine cancers (Oen); Other Skin cancers (OSk); Pancreas (Pan); Pharynx (Phx); Rectum and Anus (Rec); Salivary glands (Sal); Small Intestine (SI); Stomach (Stm); Thyroid (Thy); Tongue (Ton).

For this theme on sex differences in cancer burden, cancer researchers were invited to contribute their findings on sex differences in various cancer types across different global regions. Contributions included studies on oropharyngeal cancer (Guo et al); lung cancer (Wang et al., Park et al); brain cancer (Moss et al); and non-small cell lung cancer (Rodriguiz-Lara et al).

The study by Guo et al. highlights the significant impact of HPV vaccination on reducing Oropharyngeal Squamous Cell Carcinoma (OPSCC) incidence in the U.S. Using SEER program, the research shows a decline in HPV-related OPSCC among young adults, in both males and females, during the vaccination era. However, an increase in incidence among middle-aged and elderly vaccine-ineligible groups was observed in both males and females. Notably, cancer-specific 5-year survival improved in young males but not females, underscoring the need for further investigation. To strengthen public health messaging, the investigators conclude that efforts must be intensified to improve HPV vaccination coverage among all young females and males.

Air pollution has long been suspected to contribute to the burden of lung cancer, and recent research confirms this association (Wang et al). A study focusing on seven eastern metropolises in China sought to examine the risks and mortality associated with air pollutants such as particulate matter (PM10), nitrogen dioxide (NO2), and sulfur dioxide (SO2). From 2006-2014, decreases in PM10, NO2, and SO2 correlated with lower lung cancer rates. NO2 had the strongest association with increased lung cancer risk and mortality, highlighting the need for stricter air quality regulations. Males compared to females are thought to have a higher risk of lung cancer following exposure to ambient air pollution. The study serves as a clarion call for policymakers to intensify air quality regulations and promote cleaner environments to combat the deadly impact of air pollution on lung cancer. Another study on lung cancer from Korea found that obstructive sleep apnea (OSA) slightly reduced lung cancer risk in males but not in females (Park et al). This first-of-its-kind research analyzed a large 12-year national cohort, highlighting significant gender differences in the impact of OSA on lung cancer development.

In their sex-stratified analysis of methylation differences within medulloblastoma subgroups, Moss et al. identified sex-DMPs (Differentially methylated positions) that varied significantly, with SHH (Sonic Hedgehog) having the highest number. Notably, only SHH medulloblastoma showed sex differences in survival, with females faring worse long-term than males. They found 10 genes with conserved DMPs across subgroups, indicating a shared genetic background that may explain some of the observed sexual dimorphism. Key pathways, including TGF-β, neurotrophic receptors, and NOTCH, were implicated and may vary by sex. Importantly, four genes with sex-DMPs have available chemotherapies, suggesting potential for sex-specific treatments to improve medulloblastoma outcomes.

Rodriguez-Lara et al. presented a mini review on Non-Small Cell Lung Cancer (NSCLC) that exhibited significant differences between males and females, influenced by sex hormones. The roles of estrogen and androgen in NSCLC’s immune response remain partially understood, with contradictory data on sex-related responses to PD-L1-based immunotherapy. They point out that sex might predict NSCLC immunotherapy responses, but differences must be validated across diverse populations, considering factors like histological subtypes, mutational profiles, and smoking status. They added that females should be stratified by hormonal status, and serum hormone levels measured to clarify impacts on Programed Cell Death Ligand (PD-L1) control and immunotherapy responses. Emerging data suggest estrogen upregulates PD-L1, indicating Selective Estrogen Receptor Degraders (SERDs) could enhance immunotherapy response. Further research is crucial to understand sex-related differences, identify biomarkers, and improve therapeutic guidelines based on sex and hormonal status.

Additionally, we introduce research on methodological perspectives on measures of cancer burden using diverse datasets such as the Mortality Register System at Wuhan Center for Disease Control (Yan et al.); the Surveillance, Epidemiology, and End Results (SEER) Program (Liu et al.); the Hospital Tumor Registry at Nantong, China (Chen et al.); the U.S. National Health and Nutrition Examination Surveys (Yang et al.); Global Burden of Disease data (Zhu et al.); the National Cancer Database (Sharan et al.); and the Urban Lung Cancer Early Detection and Treatment Program in Nanchang, China (Zeng et al.).

Yan et al. showed that lung cancer deaths in Wuhan have gradually declined but aging and population growth still impact mortality rates. They concluded that reducing lung cancer mortality in Wuhan requires addressing disparities between central and surrounding urban areas. Another study included in this theme by Liu et al. using SEER Program analyzed Gastrointestinal Neuroendocrine tumors (GI-NT) cases examining incidence, survival, and risk factors. Age, stage, and pathological grade were key risk factors, with men, the elderly, and small intestine, rectum, and GI patients most affected. Race and socioeconomic status also influenced early diagnosis and treatment decisions. Chen et al. highlighted the impact of left truncation on cancer survival estimates. They point out that while hospital-based registries (HBR) evaluate prognosis, left truncation can lead to underestimation. Population-based registries (PBR) reflect overall survival but can suffer from delayed reporting, reducing survival estimates. They conclude that accurate and timely cancer registration is crucial for reliable survival data. Yang et al. presented prospective cohort study using NHANES data and found that being underweight or extremely obese increases mortality risk, primarily from cancer and cardiovascular diseases (CVD). Conversely, overweight or mildly obese conditions were linked to reduced all-cause and non-cancer, non-CVD mortality. These findings highlight the need for tailored survivorship care based on BMI. Zhu et al. highlighted the substantial lung cancer burden in Belt and Road (B&R) countries, notably China, and in South Asia, North Africa, and the Middle East. They highlight that significant gender and age differences exist, particularly in women and those over 75 years. Enhanced multi-country cooperation and policy improvements are crucial under the B&R health Initiative. Using NCDB data in the U.S., Sharan et al. identified significant disparities in treatment timelines for gastric cancer patients, influenced by age, sex, race, insurance, income, facility type, and geography. Understanding these factors is crucial for improving timely care and outcomes. They concluded that future research with updated, prospective designs will enhance strategies to address these disparities.

A screening study in Jiangxi Province, China, from 2018 to 2020, investigated low-dose computed tomography (LDCT) screening compliance among high-risk lung cancer populations (Zeng et al.). The study involved 26,588 participants, identifying 34.4% as high-risk. Screening detected suspected pulmonary tumors or lung nodules in 10.3% of patients. Better compliance was observed in males, ex-smokers, those with chronic respiratory diseases or a family history of cancer, and those with primary education. Poor compliance was linked to a history of harmful occupational exposure. These findings highlight the need to improve screening compliance by addressing these influencing factors. Li et al. in their age-period-cohort analysis showed that esophageal cancer incidence and mortality in China increased and then decreased from 1990 to 2019. They concluded that effective measures are needed to protect the elderly, who are at particularly high risk.

In conclusion, the studies presented in this editorial underscore the critical importance of addressing sex differences in cancer incidence, mortality, and survival. These sex differences offers valuable insights into cancer epidemiology, highlighting the need for robust and comparative data across regions. Research contributions reveal significant findings, such as the impact of HPV vaccination on oropharyngeal cancer, the association between air pollution and lung cancer, and the sex-specific responses to various cancer treatments. These findings emphasize the necessity for tailored public health strategies, including intensified HPV vaccination efforts, stricter air quality regulations, and sex-specific treatment approaches. Additionally, the importance of accurate and timely cancer registration, the impact of socioeconomic factors on cancer treatment, and the need for improved screening compliance are highlighted. Collectively, these studies call for comprehensive and nuanced public health policies to effectively combat the global cancer burden and improve outcomes for diverse populations.

## Author contributions

SR: Conceptualization, Writing – original draft, Writing – review & editing. WC: Writing – review & editing. AT: Writing – review & editing.

